# MRI Findings of Early Myositis Ossificans without Calcification or Ossification

**DOI:** 10.1155/2018/4186324

**Published:** 2018-09-03

**Authors:** Hexiang Wang, Pei Nie, Yang Li, Feng Hou, Cheng Dong, Yonghua Huang, Dapeng Hao

**Affiliations:** ^1^Department of Radiology, The Affiliated Hospital of Qingdao University Qingdao, Shandong, China; ^2^Department of Radiology, The Qingdao Women and Children Hospital Qingdao, Shandong, China; ^3^Department of Pathology, The Affiliated Hospital of Qingdao University Qingdao, Shandong, China; ^4^Department of Radiology, The Puyang City Oilfield General Hospital, Puyang, Henan, China

## Abstract

**Purpose:**

To characterize and evaluate the MR imaging features of early myositis ossificans (MO) without calcification or ossification.

**Methods:**

The MRI manifestations of seven patients with pathologically proven early MO were retrospectively analyzed with regard to tumor location, size, margins, signal intensity, and enhancement appearance in MR images. Additionally, the surrounding soft-tissue edema and adjacent bone change were assessed.

**Results:**

All cases (n=7) had intramuscular tumor-like masses without calcifications. The lesions appeared as isointense in T1-weighted images (T1-WI) and inhomogeneous hyperintense in T2-weighted MR images (T2-WI). On T2-WI and postcontrast T1-WI, the heterogeneously high signal intensity in the expanded muscle interspersed with a few hypointense linear structures consistent with intact muscle fibers showed “striate pattern” in the plane parallel with muscle fibers. The relatively hypointense areas with geometrical pattern consistent with the bundles of intact muscle fibers are found within the lesion with diffuse high signal intensity, displaying the “checkerboard-like pattern” in the plane vertical to muscle fibers. A “striate pattern” (n = 7) and “checkerboard-like pattern” (n = 3) in the lesion appeared in T2-WI. In contrast-enhanced MRI images, all cases showed diffuse “striate pattern” enhancement. Among them, one case demonstrated “checkerboard-like pattern” enhancement. All cases had diffuse and prominent muscle edema that preserved the muscle fascicles. For two lesions located in the deep muscle group, the adjacent bone showed bone marrow edema.

**Conclusion:**

MR imaging has unique advantages for diagnosis of early MO without calcification or ossification: the “striate pattern” and “checkerboard-like pattern” appearance shown in T2-WI and contrast-enhanced MRI images can be helpful for differential diagnosis. MRI can delineate the extent of the tumor and provides accurate anatomical information, which is important in diagnosis, treatment, and follow-up.

## 1. Introduction

Myositis ossificans (MO) is a form of solitary, benign, self-limiting, abnormal ossifying proliferation of soft-tissue mass [1]. MO may likely result from trauma, paralysis, and burns, but it may also occur with no significant history [2]. Three phases are commonly described: the early stage, which occurs within 4 weeks; the intermediate stage, seen at 4–8 weeks; and the mature stage, present at more than 8 weeks [1, 3]. In the early stage, MO without calcification or ossification is likely to be misdiagnosed as an infection or malignancy [2–4]. The mass is mainly composed of fibroblasts and myofibroblasts, with a small component of osteoid formation. It may be difficult to make a differential diagnosis between MO and sarcoma via microscopy [5]. Biopsy of heterotopic ossification (which is limited to the soft tissue called MO) may lead to further bone proliferation and result in a worsened prognosis [6].Therefore, correct diagnosis of early MO is very important, as it can help to avert unnecessary biopsy or surgery. To the best of our knowledge, there have been only a few reports about the MR imaging features of early MO without calcifications or ossification [4, 7–10], and most previous literature reports have been case reports. The authors retrospectively analyzed the characteristic MRI features of early MO to facilitate accurate diagnosis and therapeutic planning.

## 2. Materials and Methods

### 2.1. Patients

The study was approved by our institutional ethics committee. We retrospectively analyzed MRI scans obtained from seven patients (four male and three female) with pathologically proven early MO who were imaged between January 2005 and July 2017. The patients had an average age of 31.2 years (range, 17–55years).

Five of the seven patients had a history of trauma. Confirmation of an MO diagnosis required biopsy results in four patients; two patients underwent surgery; and serial radiographs were used in one. When surgery was not performed and a biopsy was not examined, the lesion was followed by CT for 2 months until it stabilized and formed a rim of well-defined cortical bone.

All seven patients underwent MRI. Contrast-enhanced T1-weighted imaging (WI) was performed in three patients, whereas CT or digital radiograph (DR) was performed in three other patients. The MRI examinations used either a 3.0-T MRI scanner (n=4; Signa HDx; GE Medical Systems) or a 1.5-T scanner (n=3; Signa Advantage Horizon; GE Medical Systems, Milwaukee, WI, USA).The following scanning parameters were used: T1-WI (500–600 ms repetition time [TR]; 15–30 ms echo time [TE], 200–400 mm field of view [FOV], 208–512 × 208–512 matrix), fat-suppressed FSE T2-WI (3600–5000ms TR; 80–120 ms TE, 200–400 mm field of view [FOV], 208–512 ×208–512 matrix), slice thickness 3–4mm, and slice spacing 1 mm. One patient underwent diffusion-weighted imaging (b=0 and 700 s/mm2), and three patients underwent contrast-enhanced MR scanning using a T1-W SE sequence (TR 500–600 ms, TE 10–15 ms) with an injection of 0.2 ml/kg gadolinium dimeglumine.

### 2.2. Imaging Analysis

Two radiologists with more than 7 years of professional musculoskeletal experience and blinded to patient history reviewed the imaging independently. The readers recorded the tumor location, size (maximum diameter of tumor), margin (well-defined or ill-defined), and signal intensity. On the plain MR images, signal intensity was classified as hypo, iso, or hyper using the adjacent muscle as reference. When contrast was administered during the MRI, the degree of enhancement was classified as none, mild, moderate, or marked. Other associated signs, including soft-tissue edema (diffuse or focal and mild, moderate, or prominent) and adjacent bone change were also recorded. The imaging findings were compared with the pathologic findings independently.

## 3. Results

Lesions were found at a variety of locations: one in the vastus intermedius muscle, one in the iliopsoas muscle, one in the tibialis anterior muscle, one fixed to the thenar muscle, one in the flexor digitorum sublimis muscle, one in the vastus lateralis muscle, and one in the brachialis and triceps muscles.

**Figure 1:**
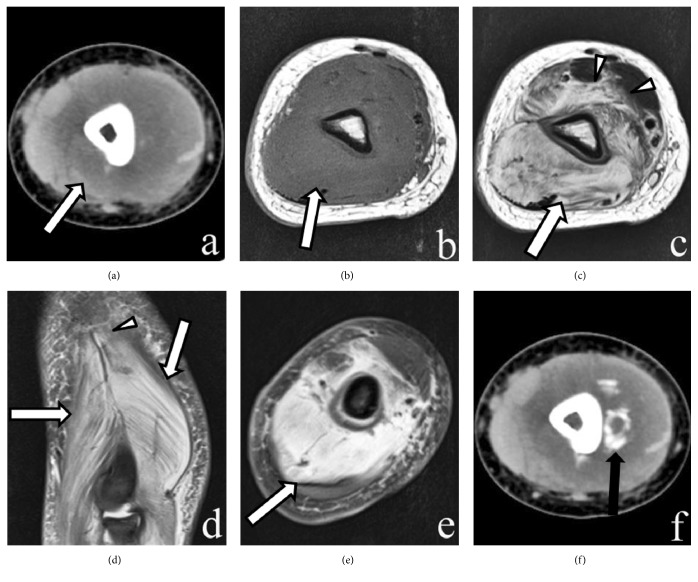
CT and MR imaging of the left forearm. (a) Axial plain CT image reveals an ovular, low-density, intramuscular mass-like lesion in the brachialis and triceps muscles. (b) Axial T1-weighted image shows an ill-defined isointense lesion. (c,d) Axial and coronal fat-suppressed T2-weighted image reveals a hyperintense lesion (large white arrow) with a “striate pattern” in the brachialis and triceps muscles with a pattern of edema in the brachialis and biceps muscles (arrowheads). (d) Enhanced fat-suppressed axial T1-weighted image shows that the lesion enhances intensely with a “striate pattern” in the muscle (large white arrow). Preservation of the muscle fascicles is noted in the lesion. (e) One month later, follow-up axial CT image shows a low-density lesion with a rim of well-defined focal and linear calcification (black arrow).

**Figure 2:**
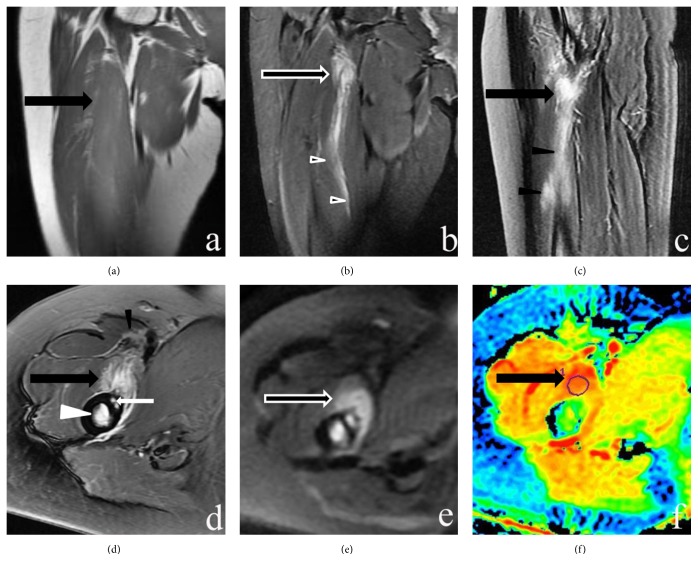
MR imaging of the right thigh. (a) Coronal T1-weighted image shows an ill-defined isointense lesion in the iliopsoas muscle (large black arrow). (b-d) Sagittal, coronal, and axial fat-suppressed T2-weighted images reveal a hyperintense lesion with a “striate pattern” in the iliopsoas muscle (large black arrow) with a pattern of edema in the iliopsoas and sartorius muscles (black arrowheads). The femur bone shows bone marrow edema (white arrowhead). The femur periosteum displays patchy areas of hyperintensity in FSE T2WI images (small white arrow). (e and f) Axial DWI and ADC mapping display hyperintensity in DWI and high ADC values of the lesion, which indicate a T2 shine-through effect.

**Figure 3:**
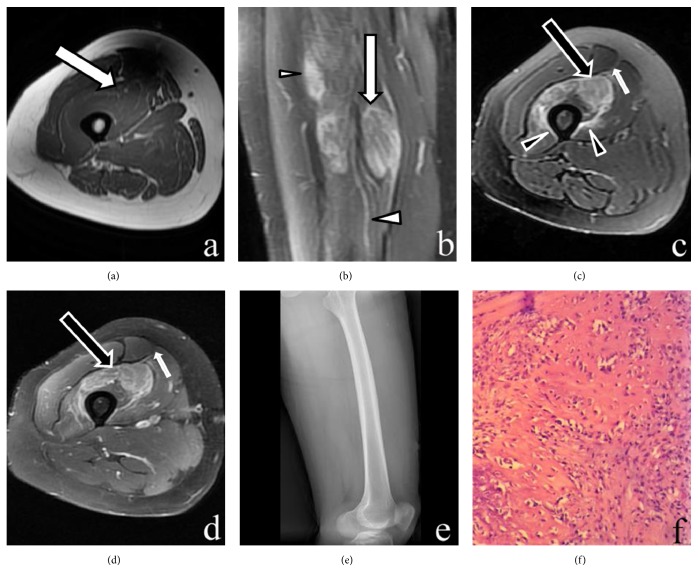
MR, DR, and histopathology imaging of the right thigh. (a) Axial T1-weighted image shows an ill-defined isointense lesion in the vastus intermedius muscle (large arrow). (b) Coronal fat-suppressed T2-weighted image reveals a hyperintense lesion with a “striate pattern” in the vastus intermedius muscle (large arrow). (c) Axial fat-suppressed T2-weighted image shows a hyperintense lesion with a “checkerboard-like pattern” in the vastus intermedius muscle (large arrow). Surrounding soft-tissue edema in the vastus intermedius and vastus medialis muscles (arrowheads) and overlying fascia (small arrow) is seen. (d) Enhanced fat-suppressed axial T1-weighted image shows that the lesion enhances intensely with a “checkerboard-like pattern” (large arrow). Preservation of the muscle fascicles is shown within the lesion. Enhancement of the overlying fascia (small arrow) is seen. (e) Lateral DR shows the anterior femoral soft tissue without any calcification or ossification. (f) The specimen mainly includes loose, immature textured fibroblasts with mild cellular pleomorphism. Some portions of the lesion contained osteoid formation. The entrapped atrophic or necrotic muscle fibers are also shown in the lesion (H&E staining, ×200).

All the cases (n = 7) were intramuscular masses without calcifications. The size of the mass ranged 8.1–49 mm (mean: 33.1 mm). All the lesions appeared as isointense in T1WI and inhomogeneous hyperintense in FSE T2WI images (Figures 1–3). All the lesions displayed ill-defined margins. On T2WI and postcontrast T1WI, the heterogeneously increased signal intensity in the expanded muscle interspersed with a few hypointense linear structures consistent with intact muscle fibers showed “striate pattern” in the plane parallel with muscle fibers. The relatively hypointense areas with geometrical pattern consistent with the bundles of intact muscle fibers are found within the lesion with diffuse increased signal intensity, appearing in the “checkerboard-like pattern” in the plane vertical to muscle fibers. A “striate pattern” (n = 7) [Figures 1(c)–1(d), 2(d), and 3(b)] and “checkerboard-like pattern” (n = 3) [Figure 3(c)] in the lesion were displayed in MR T2WI images. In contrast-enhanced MRI images, all cases (n = 3) showed diffuse enhancement with a “striate pattern” (Figure 1(e)), and, among them, one case demonstrated “checkerboard-like pattern” enhancement (Figure 3(d)). All cases showed a pattern of diffuse, prominent muscle edema that preserved the muscle fascicles (Figures 1–3). For two lesions located in the deep muscle group, the adjacent bone showed bone marrow edema (Figure 2(d)). One lesion's adjacent bone periosteum displayed patchy areas that were hypointense in T1WI and hyperintense in FSE T2WI images (Figure 2(d)). Diffusion-weighted imaging (DWI) and apparent diffusion coefficient (ADC) mapping were acquired in one patient. The lesion displayed hyperintensity in DWI and a high ADC value in ADC mapping (Figures 2(e) and 2(f)).

Histopathology showed that the lesions mainly included loose, immature textured fibroblasts with mild cellular pleomorphism. Entrapped atrophic or necrotic muscle fibers also showed in the mass (Figure 3(f)). Some small portions of the lesions had an osteoid formation component, which showed ill-defined trabeculae containing a mixture of osteoblasts, fibroblasts, and osteoid.

One month later, follow-up CT scans were examined. Plain CT showed a rim of well-defined focal and linear calcification (Figure 1(f)). Two months later, obvious calcifications were seen in the CT.

## 4. Discussion

MO is a self-limiting, benign soft tissue condition, the etiology of which is still unclear. Proposed theories about the cause of MO include hematoma ossification and connective tissue cell metaplasia [11]. The clinical presentation and radiologic findings in MO may change according to its stage of evolution. In the early stage (<4 weeks), MO mainly includes fibroblasts and myofibroblasts, with a small amount of osteoid formation [4, 11]. In the intermediate stage (4–8 weeks), MO is characterized by osteoblasts with immature osteoid formation, which gradually changes into mature bone on the periphery of the mass. In the mature stage (>8 weeks), the mass includes mature lamellar bone [1, 4, 8, 12].

MO may occur at any age, but it usually affects patients aged 20–30 years, with a slight male predominance [3]. The findings of the present study were basically similar to past findings. The patients' age ranged 17–55 years, with four of the patients presenting at age 20-30years, and the male: female ratio was 4:3. Patients with MO usually have a history of trauma, paralysis, and burns [2]: about 60%–75% of all cases result from trauma [13]. In the present study, most of the lesions (5/7) were traumatic. The clinical presentation of patients with MO differs according to the stage of progression. As in the literature [1, 5, 9], the initial symptoms were a painful, swollen mass (5/7) and decreased range of motion (3/7), but in the later stages, swelling and pain disappeared.

The radiologic findings of early MO depended on the site and extent of its evolution. According to past reports and our study, the typical MRI features of early MO are as follows: (1) the intramuscular lesion shows as isointense or slightly hyperintense in T1-WI and hyperintense in T2-WI [3, 4, 8, 14, 15], (2) the margin of the lesion may be ill-defined, with extensive surrounding muscle edema [3, 4, 16], and (3) the lesion appears diffusely or peripherally enhanced after MRI contrast enhancement [3, 4, 17]. Sometimes, periostitis, reactive joint effusions, and bone marrow edema may appear in acute conditions [3].

MRI is now regarded as the first-choice modality for early diagnosis of MO [14]. However, studies on MO have clarified that MRI findings lack specificity for MO identification [3, 15], especially in the early phase [15, 18]. In our MR images, MO presented as an intramuscular mass-like lesion with prominent surrounding muscle edema that preserved the muscle fascicles, as in the literature [4, 10]. The feature of prominent surrounding muscle edema is not usually noted in sarcomas and could be regarded as an important diagnostic feature [3]. The fact that a “checkerboard-like pattern” in the lesion was shown in axial T2WI MR images in early MO should be considered [4]. A past article [19] reported that proliferative myositis had a “checkerboard-like pattern” in MR imaging, which was in accordance with the corresponding histologic findings of fibroblastic proliferation interspersed with muscle fascicles. Early MO has been thought to have similar characteristics to this finding, primarily including proliferative fibroblasts and myofibroblasts [4]. In the present study, three lesions demonstrated those characteristics. However, all of the cases (7/7) showed a “striate pattern” in the lesion was noted in coronal and sagittal T2WI and contrast-enhanced MR images. The present cases also had fibroblastic proliferation interspersed with muscle fascicles, which usually showed at higher levels and more obviously than the “checkerboard-like pattern” in MR images. Therefore, we believe that the “striate pattern” should be the first-choice sign for diagnosis of early MO. In the present study, axial DWI and ADC mapping were acquired in one patient. The lesion displayed hyperintensity in DWI and high ADC values, indicating a T2 shine-through effect. The reason may be related to the lesion containing too much water and/or blood in the acute stage. This feature of DWI can yield detailed information about tumor composition, but further prospective studies with larger samples will be needed to confirm these findings.

Correct diagnosis of early MO is very important, as it can help to avert unnecessary biopsy or surgery. Percutaneous biopsy of heterotopic ossification, even if limited to soft tissue called MO, may lead to a worsened prognosis, and biopsy specimens can potentially be confused with osteoblastic neoplasm [6, 15]. Mature MO has typical imaging features, such as peripheral calcifications and a zonal pattern of maturation [20], which can be diagnosed very easily [4]. Therefore, instead of biopsy, repeat CT scanning is recommended in 4–6 weeks to display the evolution of zonal ossification patterns in heterotopic ossification, which is included in MO lesions [15].

Past experience indicates that the appearance of early MO without calcification or ossification in MRI may confuse radiologists and lead to misdiagnosis of the mass as sarcoma, metastasis, lymphoma, or benign conditions such as infection and proliferative myositis [9, 21]. First, the prominent surrounding muscle edema feature is not usually noted in sarcomas and could be an important diagnostic finding [3]. Moreover, in our experience, follow-up MRI showed that the prominent surrounding muscle edema of MO decreased gradually; however, in sarcoma, metastasis, and lymphoma, the edema usually does not shrink. Infectious lesions in soft tissue are usually accompanied by clinical symptoms, such as consciously significant local inflammation and hot pain with elevated counts of WBC and neutrophil granulocytes, which are effective in anti-infection treatment. In cases characterized by a rapidly growing, painful intramuscular mass-like lesion with a “checkerboard-like pattern” of muscle edema in MR imaging, early MO, or proliferative myositis should be contained in the differential diagnosis. In this condition, follow-up can be helpful for differential diagnosis [4]. During the follow-up stage, flocculent, coarse peripheral calcifications of varying thickness are noted, and obvious peripheral calcifications and a zonal pattern of maturation are seen in the mature MO. Such typical imaging features have been noted, which can make MO diagnosis very easy [4].

In conclusion, MO has some specific features in MRI imaging, such as the “striate pattern” and “checkerboard-like pattern” appearance. These features can give detailed information about tumor composition and be helpful for differential diagnosis. Although ossification is not visible, early MO should be considered, particularly in traumatic, rapidly growing, painful lesions with prominent muscle edema in MR imaging.

## Data Availability

The data used to support the findings of this study are available from the corresponding author upon request.
